# Effectiveness of the single‐shot dual‐energy subtraction technique for portal images

**DOI:** 10.1120/jacmp.v12i4.3232

**Published:** 2011-11-15

**Authors:** Hideki Fujita, Sakon Morimi, Michihiro Yamaguchi, Haruyuki Fukuda, Kenya Murase

**Affiliations:** ^1^ Department of Radiation Oncology Osaka Saiseikai Nakatsu Hospital Osaka; ^2^ Department of Medical Physics and Engineering, Division of Medical Technology and Science, Course of Health Science, Graduate School of Medicine Osaka University Osaka; ^3^ Department of Radiology Osaka Prefectural Medical Center for Respiratory and Allergic Diseases Osaka Japan

**Keywords:** single‐shot dual‐energy subtraction, portal image, storage phosphor plate, bone‐enhanced image

## Abstract

The aim of the present study was to evaluate the clinical efficacy of the single‐shot dual‐energy subtraction technique for obtaining portal images. We prepared two storage phosphor plates for this study. A 1 mm thick tungsten sheet was placed between the two storage phosphor plates. A single use of the double‐exposure technique provides two portal images simultaneously (i.e., a standard image and a low‐contrast image), using the same patient position and with no additional radiation delivered to the patient. A bone‐enhanced image is created by image subtraction between these two images. For evaluation of clinical efficacy, three treatment sites — the brain, lung, and pelvis — were imaged. Ten sets of images were obtained for each site, and five landmarks were selected for each treatment site. The visibility of each landmark and the ease of overall verification for the selected treatment sites were assessed separately for the standard and bone‐enhanced images. Four observers consisting of one radiation oncologist and three radiation therapists participated in the present study. For most of the landmarks studied, the bone‐enhanced images were significantly superior to the standard images. Regarding the ease of overall verification, the bone‐enhanced images were significantly superior to the standard images at all sites. The p‐values of mean rating for the brain, lung, and pelvis were 0.002, 0.012, and 0.003, respectively. The bone‐enhanced images obtained using our technique increased the image quality in terms of bone visibility, and are considered useful for routine clinical practice.

PACS number: 87.56.Da

## I. INTRODUCTION

In external beam radiotherapy, patient setup verification is very important and is verified by portal images. There are several portal imaging systems for verification of patient setup (i.e., conventional portal films, digital computed radiography (CR) systems, and electronic portal imaging devices (EPID)).^(^ ^1^ ^–^ ^7^ ^)^ Recently, conventional portal film methods have been replaced by digital systems and are not commonly used. CR systems using a storage phosphor plate are still used for portal imaging in many institutions because they have the advantage that they can be used in several treatment rooms and simulators at a significantly lower cost than EPID. On the other hand, EPID using a flat‐panel detector has advantages over CR systems. For example, the images are available immediately and can therefore be used interactively to adjust the patient or field position during treatment. Recently, linear accelerators have been equipped with EPID, which are currently used widely.

The quality of portal image affects the accuracy of verification. When the quality of a portal image is improved, the margins around the clinical target volume in the definition of the planning target volume are minimized, contributing to the provision of high‐quality treatment. The bony anatomy is most important as a reference structure, and bone visibility for portal verification needs to be improved. Several techniques have been devised to improve image quality. One example is flexible noise control image processing for CR portal imaging.^(^ ^3^ ^)^ Although this method is helpful for verification because of the reduced image noise, the image quality of the bony anatomy is insufficiently improved. The quality of the portal images obtained by EPID is superior to that of the images acquired by CR due to improvement of the detector and the optimization of acquisition parameters.^(^ ^5^ ^–^ ^7^ ^)^ However, the resolution of the anatomical structures requires further improvement to enable more precise verification.

With the aim of improving the image quality in terms of bone visibility, we focused on the dual‐energy subtraction technique, which is routinely used in the diagnostic field.^(^ ^8^ ^–^ ^11^ ^)^ With this technique, the soft‐tissue and bone images are created by image subtraction between two digital images obtained with two different X‐ray spectra. Several investigators have reported that soft‐tissue images could help enhance detection of pulmonary nodules and calcifications in dual‐energy subtraction chest radiography or mammography.^(^ ^8^ ^–^ ^11^ ^)^ Two approaches (i.e., a single‐shot technique and a dual‐shot technique) have been developed and evaluated by some research groups. In the single‐shot technique, a single X‐ray exposure is used to expose two stacked detectors, which may be separated by a filter.^(^ ^8^ ^,^ ^9^ ^)^ The dual‐shot exposure technique employs two separate exposures: a high‐energy exposure and a low‐energy exposure, which are subsequently applied to the same detector.^(^ ^10^ ^,^ ^11^ ^)^ Since the dual‐shot technique requires additional radiation, we employed the single‐shot technique for the present study.

A metal plate is usually required in front of the digital imaging detector for acquisition of portal images because secondary electrons generated by the metal plate produce portal images of better quality.^(^ ^12^ ^,^ ^13^ ^)^ When the digital imaging detector is not equipped with a metal plate, the quality of the portal image in terms of bony visibility is reduced.^(^ ^4^ ^)^ Accordingly, subtraction of the two images with and without a metal plate may produce a bone enhancement image. In this study, we investigated the clinical efficacy of the single‐shot dual‐energy subtraction technique for portal images in order to improve bone visibility.

## II. MATERIALS AND METHODS

### A. Image acquisition

**Figure 1:**
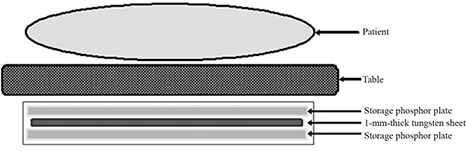
Cross‐sectional representation of a combination of two storage phosphor plates and a metal plate for acquisition of two portal images. The metal plate (1 mm thick tungsten sheet) is placed between two storage phosphor plates. The low‐contrast image is generated from the front storage phosphor plate without a metal plate. The high‐contrast image is obtained from the back imaging detector with a metal plate. Two portal images are simultaneously obtained by a single use of the double‐exposure technique.

None of the currently commercially available EPID can be used for our study because they do not enable removal of the metal plate from the imaging detector. We therefore used a CR system in this study. The storage phosphor plate, which is the imaging detector of the CR system, enables removal of the metal plate.^(^ ^4^ ^)^ We prepared two storage phosphor plates for capturing the single‐shot dual‐energy subtraction portal images. Figure 1 shows the arrangement of the two storage phosphor plates and the metal plate. The CR system used in the present study was a REGIUS Model 150 (Konica Minolta Holdings, Inc., Tokyo, Japan). For the metal plate, a sheet of tungsten measuring 1 mm in thickness, which is used in routine clinical practice, was used.

**Figure 2:**
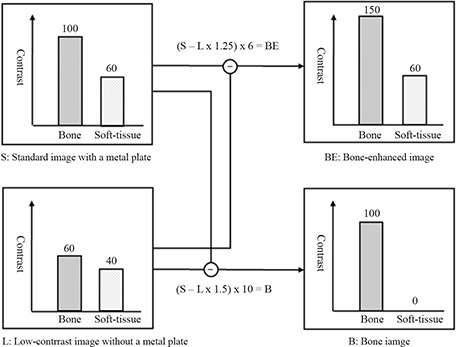
Theory behind the dual‐energy subtraction technique. The bone image is generated by correcting the soft‐tissue contrast of image L to be equal to that of image S and emphasizing bone contrast. Similarly, the bone‐enhanced image is created by adjusting image L and image S to the optimal conditions based on visual judgment. When the contrast of bone and the contrast of soft‐tissue are the numbers shown in this figure, the two equations provide the bone‐enhanced image and the bone image. In reality, however, the contrast of bone and the contrast of soft‐tissue do not give a certain number, and in addition, the contrast varies depending on the images.

A single use of the double‐exposure technique provides two portal images simultaneously with the same patient position and no additional radiation to patients. The front storage phosphor plate without a metal plate generates a portal image of low contrast. A portal image of high contrast is obtained using the back storage phosphor plate with a metal plate (i.e., a standard image). The bone image is obtained by image subtraction between the standard and low‐contrast images. Actually, a bone‐enhanced image including both soft‐tissue and bone is required, because the soft‐tissue image is required for verification of the setup position. The theory behind the dual‐energy subtraction technique is shown in Fig. 2.

The linear accelerator used was an MHCL 15DP (Varian Medical Systems, Tokyo, Japan). The nominal photon energy was 4 MV, and the source–isocenter distance was 100 cm. Portal images were obtained using the double‐exposure technique and required four monitor units (MU) using LG (linac graphy) mode, in which each exposure was 1 MU in the treatment field with the multileaf collimator (MLC), and the second exposure was 3 MU outside the radiation field without the MLC and X‐ray jaws being opened. The dose in the LG mode is a factor of 10 lower than that in the treatment mode, so 1 MU in the LG mode is equivalent to 0.1 MU in the treatment mode.^(^ ^3^ ^)^ The CR device we used provides portal images at 1 MU or lower. In the treatment mode, however, exposure cannot be performed at 1 MU or lower. In order to minimize additional dose associated with portal images, LG mode is used when portal images are obtained. After irradiation, the two storage phosphor plates were scanned, and the 12‐bit digital data were processed by the CR system. The image processing parameters of the CR system that we reported earlier^(^ ^14^ ^)^ were employed to process the image data of the two storage phosphor plates. Image processing parameters of the CR system were adjusted in order to acquire optimal portal images, which included gradation processing and frequency processing. Contrast and brightness of portal images were set at the optimal image processing parameters. The two portal images obtained were archived to an online image server (POP‐Net Server, ImageOne Co., Ltd, Tokyo, Japan). A pair of portal images was imported to a personal computer from the image server. Image processing for the dual‐energy subtraction technique was performed on a personal computer under the optimal processing conditions based on visual judgment by a medical physicist (operating system: Windows XP, Microsoft Corp, Redmond, WA, USA).

### B. Image evaluation

Three treatment sites (i.e., the brain, lung, and pelvis) and 30 sets of images (10 sets per site) were used to assess clinical efficacy. Five landmarks, which are typically used by radiation oncologists for portal verification, were chosen for each treatment site. These landmarks are shown in Table 1. The image data of the three treatment sites were accumulated sequentially during regular patient treatments. Each set, consisting of a standard portal image or a bone‐enhanced image and its corresponding simulation image, was randomly displayed on a viewing monitor (RadiForce G31, EIZO NANAO Corporation, Ishikawa, Japan). The simulation images were digitally reconstructed radiographs (DRR), which were produced using a CT simulator and treatment planning system. The CT simulator used was an Emotion Duo scanner (Siemens Medical Solutions, Forchheim, Germany), and the CT images were acquired at a detector row width of 2.5 mm, volume pitch of 3.0, 3.0 mm section thickness and intervals, 0.8 second rotation time, and 120 kV, using the automatic tube current modulation technique. The CT images were transferred to XiO treatment planning system (CMS JAPAN, Tokyo, Japan), and the DRR were produced by XiO.

**Table 1:** Mean rating of four observers for each landmark at three treatment sites.

*Site*	*Landmark*	*Bone‐enhanced Mean (SD)*	*Standard Mean (SD)*	*p‐value*
Brain	Peripheral skin	1.5 (0.51)	1.6 (0.50)	0.157
	Skull brim	1.2 (0.42)	1.4 (0.50)	0.011
	Mastoid air cells	1.9 (0.52)	2.1 (0.60)	0.020
	Frontal sinus	1.7 (0.47)	1.9 (0.47)	0.002
	Vertebral bodies	2.9 (0.70)	3.1 (0.55)	0.005
Lung	Lung airspace	2.0 (0.48)	2.0 (0.53)	0.527
	Trachea	2.3 (0.47)	2.6 (0.50)	0.004
	Tumor	1.7 (0.48)	1.9 (0.43)	0.021
	Ribs	1.9 (0.52)	2.1 (0.45)	0.035
	Vertebral bodies	2.8 (0.69)	3.1 (0.75)	0.012
Pelvis	Acetabulum	1.8 (0.50)	2.0 (0.53)	0.011
	Femoral head	1.9 (0.40)	2.1 (0.32)	0.035
	Pelvic brim	2.3 (0.46)	2.5 (0.55)	0.020
	Sacroilliac joint	2.4 (0.48)	2.6 (0.55)	0.013
	Pubic symphysis	2.4 (0.48)	2.5 (0.51)	0.025

Bone‐enhanced=bone‐enhanced portal image; Standard=standard portal image

The four observers who participated in the study consisted of one experienced radiation oncologist and three radiation therapists with at least five years of radiotherapy experience each. They were unaware of the type of image‐processing technique used. The observers were allowed to adjust contrast, brightness, or window width and level of the images. We required a minimum interval of a week between the two interpretation sessions. We added explanation of standardization to the manuscript. Without a viewing time limit, each observer evaluated a portal image on the viewing monitor and gave a rating in response to two questions.

The first question was concerned with the visibility of each landmark and was answered separately for the standard and bone‐enhanced portal images:

Based on the information derived from the DRR and a portal image (the standard image or the bone‐enhanced image), the individual landmark in the portal image was: 1) very clear, 2) clear, 3) visible, 4) not clear, or 5) not visible.

The second question was related to the ease of overall verification of the treatment areas, which was assessed separately for the standard and bone‐enhanced portal images:

Based on information derived from the DRR and a portal image (the standard image or the bone‐enhanced image), the overall verification decision was: 1) very easy, 2) easy, 3) possible, 4) difficult, or 5) very difficult.

The study design was determined in reference to the methods described by Yin et al.,^(^ ^15^ ^)^ and Yamada and Murase^(^ ^3^ ^)^ who conducted an observer study on the direct comparison of clinical efficacy.

Data were expressed as the mean ratings and standard deviation (SD) of the observers. We used the Wilcoxon signed rank test for comparisons between two images with the significance level set at p<0.05. The statistical analyses were performed using SPSS 11.0 for Windows (SPSS Inc., Chicago, Illinois, USA).

## III. RESULTS

**Figure 3:**
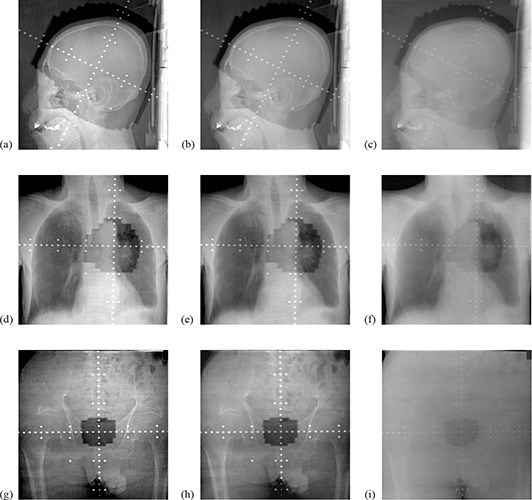
Samples of bone‐enhanced and standard images: (a), (b), (c) show the head; (d), (e), (f) show the chest; (g), (h), (i) show the pelvis. Note: figures (a), (d), (g) are the bone‐enhanced images, figures (b), (e), (h) are the standard images, and figures (c), (f), (i) are the low‐contrast images.

**Figure 4:**
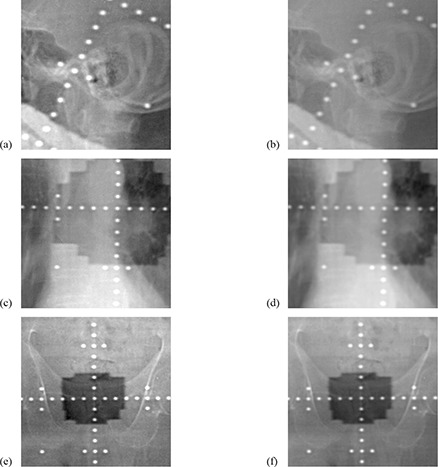
Parts of the bone‐enhanced images and standard images after magnification: (a), (b) show a part of the magnified head; (c), (d) show a part of the magnified lung; (e), (f) show a part of the magnified pelvis. Note: figures (a), (c), (e) are the bone‐enhanced images, and figures (b), (d), (f) are the standard images.

Figure 3 shows the bone‐enhanced portal images on the left, their corresponding standard images in the center, and low‐contrast images on the right. Figure 4 shows parts of the images after magnification. The image quality of the bone anatomy, such as the bone brim, was improved by the single‐shot dual‐energy subtraction technique, but the image quality of the soft tissue, such as the skin and airspace, was not improved because the image noise was increased.

Mean ratings for the observer study in respect of the image quality for each landmark are shown in Table 1. For most of the landmarks studied, the bone‐enhanced portal images were significantly superior in quality to the standard images. For the peripheral skin and lung airspace, however, the visibility was comparable between the two images, giving no significant differences (p=0.157 and 0.527, respectively). The results for the ease of overall verification of the treatment areas are shown in Table 2. The bone‐enhanced portal images were significantly superior to the standard images at all sites, and the p‐values for the sites of brain, lung, and pelvis were 0.002, 0.012, and 0.003, respectively.

**Table 2:** Mean rating of four observers for ease of the overall verification at each treatment site.

*Site*	*Bone‐enhanced Mean (SD)*	*Standard Mean (SD)*	*p‐value*
Brain	1.3 (0.45)	1.5 (o.51)	0.002
Lund	1.5 (0.51)	1.8 (0.45)	0.012
Pelvis	2.1 (0.60)	2.4 (0.48)	0.003

Bone‐enhanced=bone‐enhanced portal image; Standard=standard portal image

## IV. DISCUSSION

We have developed a portal imaging technique involving single‐shot dual‐energy subtraction. Generally, the high‐contrast image is obtained from the front imaging detector without a filter, and the low‐contrast image is generated from the back imaging detector with a filter. However, in our technique, the high‐contrast image is obtained from the back imaging detector with a filter because the metal plate used as a filter acts as an intensifying screen. The dual‐energy subtraction technique used in the diagnostic field utilizes a difference in contrast which is produced by a difference in energy. It is considered that at a high‐energy level, the change of spectrum is small and the resulting difference in contrast due to the difference in energy is minimal. In this study, we used a 1 mm thick tungsten sheet as a filter. Kihlén et al.^(^ ^16^ ^)^ reported that a metal with an atomic number around 26–29 would be an optimal material for a metal screen with regard to quality after measuring the scattered to primary dose ratio. On the other hand, Kausch et al.^(^ ^17^ ^)^ reported that metals with a high atomic number perform better than lighter metals in maximizing the detective quantum efficiency using Monte Carlo simulation. In the future, investigation of the optimal thickness or material of a filter to be used would further improve the image quality of both standard and bone‐enhanced portal images.

According to the observer study results for the visibility of landmarks, most of the observers concluded that the bone‐enhanced portal images were equal to or more clear than the standard images in terms of bony anatomy. However, the image quality was almost comparable between the two landmarks, giving no significant differences. The dual‐energy subtraction technique is primarily intended to enhance the outline of bones, and it is difficult to improve the image quality in all treatment areas. Although the trachea and tumors are not bones, the bone‐enhanced images of then were superior to the standard images. This may be explained by the enhancement of the edges of the trachea and tumors, which facilitated recognition of their outlines. In all landmarks for the pelvis, significant differences were noted between the two images. These differences may be particularly useful, because the bony anatomy is typically used as reference structure for setup verification of the pelvis.

The results for the ease of overall verification indicated that bone‐enhanced portal images obtained with the energy subtraction technique are superior to standard portal images. The most important function of the portal image is to evaluate the radiation field position based on a comparison with a simulation image, and the bone visibility is of most importance for this. Therefore, we consider that observer judgment is not influenced much by the slight increase in noise. In any case, we expect that the image quality will be improved, because bony anatomy is used as the reference structure in many sites. These results indicate that bone‐enhanced portal images are of sufficiently high quality for clinical application and may be used clinically instead of standard images. A standard image provides the base of our imaging methodology. Accordingly, even though we make the best use of our technique, we cannot obtain substantially greater information. However, if bone visibility is improved only slightly, then the accuracy of visual inspection or of verification in auto‐registration would be improved, shortening the time required for the verification. Significant differences were noted between the two types of images (i.e., standard images and images obtained by our technique) in terms of how clearly we can see the bones. Therefore, we consider that our technique is clinically useful.

There are some limitations in the technique presented. In general, the effect produced by the dual‐energy subtraction technique strongly depends on the spectral separation between the low‐ and high‐energy images. However, even if we use an optimal metal in high‐energy radiation therapy, it is difficult to produce differences and the effect is insufficient with X‐ray absorption. In addition, the image noise is increased by addition of the noise components of the two images. Although the influence of noise on image quality was limited in the four sites and each landmark of our observer study, the noise component may affect portal verification at other sites or landmarks. Another limitation is that the processing conditions were determined on the basis of visual judgment. The optimal conditions were different at all sites and depended on a medical physicist. The purpose of our study was to confirm the effectiveness of a single‐shot dual‐energy subtraction technique. For this purpose, we first adjusted image processing conditions so as to increase visual clarity of bone anatomy. When the numbers for the contrast of bone and soft‐tissue are identified as in Fig. 2, we can identify an optimization algorithm (i.e., the equation). In real images, however, the contrast does not give any specific number and it is therefore difficult to determine the equation. For example, when we try to identify a mask radius and a mask weight in the case of unsharp masking technique, what we do generally is to explore optimal processing conditions based on visual judgment. A computer algorithm to provide the optimal processing conditions needs to be developed in the future. If an objective optimization algorithm were clarified, we could obtain a better bone‐enhanced image.

In our technique, the difference in contrast between the two images obtained using the two plates is related to how scattered electrons emitted from the tungsten plate act. Low‐contrast images obtained by our technique are similar to those obtained by the unsharp masking technique. Namely, our image processing technique is similar to the image processing method of the unsharp masking technique. However, these two techniques differ as follows: the unsharp masking technique produces grains or noise on images which are also emphasized, whereas our technique minimizes noise. In the future, we plan to evaluate contrast and resolution of the images through image quality assessment.

We used a storage phosphor plate, because none of the commercially available EPID are appropriate for the single‐shot technique at present, since removal of the metal plate is required. Our goal is to improve the image quality using the dual‐energy subtraction technique with the EPID. The standard image acquired using flat‐panel detector is superior to the image obtained using storage phosphor plates. Therefore, the bone‐enhanced image created using flat‐panel detectors may further improve image quality. In future, we intend to demonstrate that the bone‐enhanced image created using flat‐panel detectors is useful for clinical practice.

## V. CONCLUSIONS

We have developed the single‐shot dual‐energy subtraction technique for portal images, and evaluated its clinical efficacy. The bone‐enhanced portal images obtained using our technique contributed to the improvement of image quality in terms of bone visibility, and were superior to standard portal images. These results indicate that the bone‐enhanced portal image is useful in routine clinical practice. We conclude that our technique has the potential to improve the image quality of portal images.
